# Retracted: Effect of Moderate-Intensity Aerobic Exercise on Hepatic Fat Content and Visceral Lipids in Hepatic Patients with Diabesity: A Single-Blinded Randomised Controlled Trial

**DOI:** 10.1155/2023/9829387

**Published:** 2023-10-10

**Authors:** Evidence-Based Complementary and Alternative Medicine

*Evidence-Based Complementary and Alternative Medicine* has retracted the article titled “Effect of Moderate-Intensity Aerobic Exercise on Hepatic Fat Content and Visceral Lipids in Hepatic Patients with Diabesity: A Single-Blinded Randomised Controlled Trial” [1], due to concerns regarding redundancy, the standard of reporting, and suitability of the statistical analysis.

Concerns with this article have been raised in a Statement of Concern [2] and Letter to the Editor [3] published in *Medicine* by Dr. Colby J. Vorland, Greyson Foote, Stephanie L. Dickinson, Dr. Evan Mayo-Wilson, Dr. David B. Allison, and Dr. Andrew W. Brown. Following assessment by the journal and the editorial board, the following issues have been identified with this article:

- There is no mention of the clinical trial registration number, NCT03774511 (retrospectively registered in December 2018), or that this was part of a larger study. Overall, there were three arms: a control, a high-intensity exercise group (HII) and a moderate-intensity exercise group (MIC), but only the control and MIC were reported in [1].
- There is no indication that references 35 and 36 [4, 5] cited in the article draw on data from the same study participants and these references are incorrectly presented as separate studies supporting the findings of the article, which may have misled readers.
- The authors have stated that recruitment and randomization occurred during August-December 2017, the HII and control arms were conducted during January-August 2018, and the MIC arm was run during August-December 2018, which is a non-standard study design and was not reported in any of the articles.
- The data presented in Figure 1 and Tables 1 and 2 are identical to data presented in Abdelbasset et al. [5]. With respect to Figure 1 the study has been presented without the additional study arm shown in Abdelbasset et al. [5].
- The data in Table 2 is identical to that shown as the MIC study arm in Abdelbasset et al. [5]. However, the *p* values have been presented to three decimal places whereas in Abdelbasset et al. [5] they are presented to two decimal places [5]. The data also shows inconsistent rounding. There is a particular concern where 0.046 has been rounded down to 0.04 (and hence appears statistically significant) rather than rounding up, as has occurred with other values. In addition, several items shown as *p*=0.01 in Abdelbasset et al. [5] are shown as values less than 0.01 (i.e., <0.01, 0.004 and 0.002).
- There are concerns with the accuracy of the statistical tests reported in the article, because the comparisons are of within-group differences rather than using valid between-group tests such as ANOVA. Many of the *p*-values reported in the article could not be replicated by Vorland et al. [3], and in particular they found no significant differences between treatment groups for BMI, IHTG, visceral adipose fat, total cholesterol, and triglycerides. This was confirmed by the authors' reanalysis, apart from triglycerides for which there was a significant difference between treatment groups according to the authors' reanalysis.
- The age ranges are slightly inconsistent between the articles, despite the studies collectively reporting on the same participants: 45–60 in [1, 4] and 40–60 in [5]. The authors state that 40–60 years reflects the inclusion criteria for the study, whereas the actual age range of the included participants was 45–60 years.
- Although this was a single clinical trial, different ethical approval numbers are given in each article: PT/2017/00-019 [1], PT/2017/00-018 [4], and P.TREC/012/002146 [5].

The authors do not agree to the retraction and the notice.
